# Successive Generations in a Rat Model Respond Differently to a Constant Obesogenic Environment

**DOI:** 10.1371/journal.pone.0129779

**Published:** 2015-07-01

**Authors:** Alice H. Tait, David Raubenheimer, Mark P. Green, Cinda L. Cupido, Peter D. Gluckman, Mark H. Vickers

**Affiliations:** 1 Liggins Institute and Gravida: National Centre for Growth and Development, University of Auckland, Auckland, New Zealand; 2 Institute of Natural and Mathematical Sciences, Massey University, Albany, Auckland, New Zealand; 3 The Charles Perkins Centre and Faculty of Veterinary Science and School of Biological Sciences, The University of Sydney, Sydney, New South Wales, Australia; 4 School of BioSciences, University of Melbourne, Melbourne, Victoria, Australia; Monash University, AUSTRALIA

## Abstract

Research has shown that if a mother experiences a transitory perturbation to her environment during pregnancy or lactation, there are transgenerational consequences often involving a disordered metabolic phenotype in first generation offspring with recovery across subsequent generations. In contrast, little is known about the nature of the transgenerational response of offspring when a mother experiences a perturbation that is not transitory but instead persists across generations. Our study, using a rat model, subjected the parental generation to a change in environment and concomitant shift from a grain-based to obesogenic diets to generate an adipose phenotype in first generation offspring emulating a common scenario in human urbanisation and migration. We then investigated whether the obese phenotype was stable across generations when maintained in the transitioned environment, and whether dietary macronutrient balance affected the response. We found that second and third generation offspring had a reduced body fat to lean mass ratio and a reduced appetite relative to first generation offspring, irrespective of dietary macronutrient balance. The trajectory of this response is suggestive of a reduction in chronic disease risk across generations. This is one of the first studies, to our knowledge, to investigate the transgenerational response following parental transition to a persistent obesogenic environment, and to demonstrate that successive generations respond differently to this constant environment.

## Introduction

Epidemiological and experimental studies in animal models have shown that susceptibility to developing chronic disease in offspring may be increased if the mother experienced a transitory nutritional perturbation during pregnancy or while nursing, for example a period of calorie restriction, protein restriction or high fat feeding [1–8]. This phenotype is expressed even though the offspring themselves experienced no direct manipulations and were normally-nourished. Animals studies have further shown that second generation offspring may also be susceptible to developing chronic disease as a result of the transitory nutritional perturbation experienced by their grandmother, though third generation offspring show no such tendencies [9–16]. This transgenerational response is thought to be underpinned by mechanisms of epigenetic inheritance [17, 18].

In contrast to the body of data on offspring responses following a transient change in maternal environmental conditions, little is known about the response following a sustained transition in environmental conditions, which is first encountered by the parent then persists across generations. This scenario is highly relevant to the human nutritional transition. In human society, globalization involving global trade liberalization and increases in income and urbanization has created obesogenic environments by promoting positive energy balance through: (a) nutritional transitions to diets rich in animal products, refined grains and added sugar; and (b) reductions in physical activity [19–23]. Populations worldwide are transitioning in this way, and are continuing to experience obesogenic environments across generations. This has seen the worldwide prevalence of obesity double between 1980 and 2008 [19, 24]; whether or not this trend will persist in the face of on-going obesogenic environments is of much interest.

In this study, using a rat model, we manipulated the environment of the parents to emulate the human scenario of migration from rural to urban environments, or from a low to a high income society/country. This scenario involves both a radical change in environment and a change in nutrition to more obesogenic diets, which in humans leads to a greater risk of obesity[25, 26]. Our parental generation of rats simultaneously experienced a change in environment by way of relocation from the supplier’s facility to our research facility, and a change from a grain-based diet to a purified diet, akin to the shift towards more refined diets in humans. We then recorded the body composition and food intake of first, second and third generation offspring maintained under constant conditions on the transitioned diet. To investigate whether dietary macronutrient balance affected the transgenerational response, we also introduced a high fat or a low protein purified-ingredient diet to a subset of first generation offspring, whose descendants were likewise fed the high fat or low protein diets.

## Methods

### Experimental design

**Fig 1:**
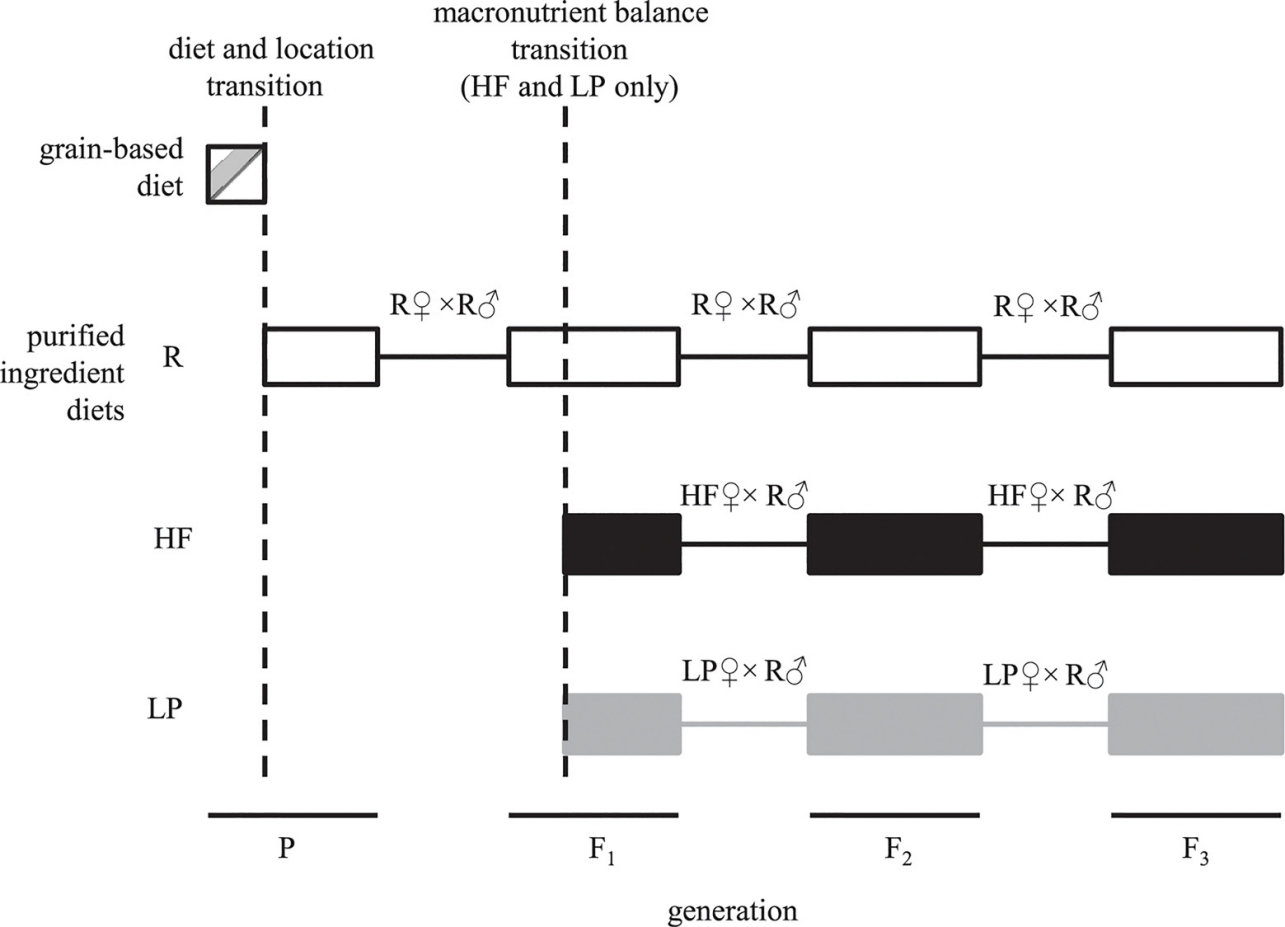
Experimental design. The parental generation of rats (P) experienced a transition aged approx. 32 days in the form of relocation to our animal facility and a shift from grain-based nutrition to a reference purified-ingredient diet (R). P rats were mated to produce the first of three generations fed exclusively on purified-ingredient diets (F_1_). F_1_ offspring were either maintained on R, or were fed a high fat purified diet (HF) or a moderately low protein purified diet (LP) from weaning onwards. Second and third purified diet generations (F_2_, F_3_) were generated by mating females from each lineage (R, HF, LP) with males from the R lineage.

The parental generation (P) of Wistar rats (15 males, 30 females, all non-littermates) was sourced from a colony maintained on a grain-based diet (VRF1) from Charles River Laboratories (Charles River UK Ltd., Kent, UK) and transported to our research facility in Auckland, New Zealand. Upon arrival, aged approximately 32 days, P rats experienced a nutritional transition to a reference purified-ingredient diet (R) (Table 1, Fig 1). Purified-ingredient diets can be obesogenic for rats due to increased palatability, greater energy density [27, 28] and greater nutrient conversion efficiencies [27, 29, 30]. First, second and third generation offspring of P rats (F_1_, F_2_ and F_3_, respectively) were all fed exclusively on purified-ingredient diets.

**Table 1:** Diet composition.

Diet		metabolizable energy (kcal/g)	protein (% kcal)	carbohydrate (% kcal)	fat (% kcal)
grain-based		3.3	24	63	14
purified-ingredient	reference	3.8	19	69	12
	high fat	4.7	18	35	46
	low protein	4.1	12	66	22

To investigate whether or not the macronutrient balance of the purified-ingredient diets influenced the transgenerational response, in addition to the lineage maintained in R a subset of F_1_ rats was weaned onto either a high fat purified-ingredient diet (HF) or moderately low protein purified-ingredient diet (LP) (Table 1) (F_1_ litters were systematically distributed among the three diet treatment groups to avoid litter biases among the dietary treatment lineages). F_2_ and F_3_ offspring were generated on each dietary lineage (R, HF and LP) by mating females from each lineage with unrelated males of the same generation from the R lineage. All purified-ingredient diets were fed *ad libitum* and were supplied by Purina TestDiet (PMI Nutrition, Richmond, IN, USA; R = 58B0 (AIN-76A); HF = 58V8 (D12451); LP = 5A3U (modified AIN-76A)).

### Animal model

All animal procedures were approved by the Animal Ethics Committee of the University of Auckland (Approval R402). Rats were housed from weaning in pairs or as singletons during pregnancy under standard conditions with wood shavings for bedding and free access to water in a room with a 12 h light / 12 h dark cycle, a temperature of 25°C and humidity of 50%. F_1_, F_2_ and F_3_ rats were housed in the same room and all procedures including daily husbandry were conducted by the same team of personnel throughout the study.

Virgin females were time-mated at approximately 130 days of age, using an oestrous cycle monitor (Model EC40, Fine Science Tools, Foster City, CA, USA) to assess the stage of oestrous before introducing the male, and housed singly after pregnancy confirmation. At birth, the sex and weight of each pup were recorded but litters were left intact until postnatal day 2 when they were standardised to 8–10 pups (equal number of males and females) to remove potential litter size effects (litters of 8–10 pups were left intact while litters of >10 pups were standardised to 10 pups by randomly selecting and then culling excess pups). At 22 days of age, pups were weaned from their mothers and housed in same-sex sibling pairs. From weaning until the end of the study, animals were checked daily and their bodyweights recorded every six days to monitor general wellbeing. Details of the animal model including matings, births and neonatal survival are presented in S1 Table.

### Body composition, calorie intake and nutrient conversion efficiencies

Body composition was measured on a subset of eight males and eight females per group at approximately 115 days of age. This was done under light halothane anaesthesia (3%) by dual-energy X-ray absorptiometry (DXA) with dedicated small animal software (GE Medical Systems Lunar, Madison, WI, USA). Food (calorie) intake was measured across consecutive six-day periods from weaning onwards. We report the average intake of rats from age 58 days onwards (across nine consecutive periods) over which time intake was relatively stable. Since rats were housed in pairs, food intake was measured per pair and meaned to estimate the intake per rat. Nutrient conversion efficiencies were also evaluated per pair of rats with ANCOVA to analyse the combined body lean or fat mass of each pair, using their combined average daily intake of protein or non-protein energy, respectively, as the covariate [31, 32].

### Data analysis

Data from F_1_, F_2_ and F_3_ rats were analysed by two-way ANOVA or ANCOVA using generation and diet as factors, where a significant main effect of generation indicates that the phenotype was not stable (*i*.*e*. non-consistent) across generations, a significant statistical interaction between generation and diet indicates an effect of macronutrient balance on the transgenerational response, and a significant main effect of diet indicates a within-generation effect of diet. Sample sizes are presented in S2 Table. Male and female data were analysed separately based on the *a priori* assumption that the sexes may respond differently. Body composition and calorie intake data were analysed by two-way ANOVA and food conversion efficiency data were analysed by two-way ANCOVA with nutrient intake as the covariate (protein and non-protein energy as the covariate for body lean and fat mass, respectively) using SPSS (PASW Statistics 18, IBM Corp., Somers, NY, USA). Homoscedascity was confirmed with Levene’s test with a square root or rank transformation applied as necessary. Where the statistical interaction between generation and diet was significant (*p* < 0.05), the analysis was split by diet and the effect of generation was tested for each diet using one-way ANOVA or ANCOVA as appropriate. Otherwise, the interaction term was removed before testing the main effects. Where appropriate, for ANOVA Scheffe analysis was used for *post-hoc* tests of generation and Dunnett’s analysis with R as the control group for *post-hoc* tests of diet, and for ANCOVA the main effects were compared using a Sidak correction. Litter identity was initially included as a random effect in a mixed model ANOVA (using SAS version 9.1.3 for windows software, SAS Institute Inc., Cary, NC, USA), but there were no qualitative differences between the results of the standard model and this mixed model ANOVA, so litter identity was excluded to simplify the analysis and presentation. Data are presented as means ± SEM for ANOVA and estimated marginal means ± SEM for ANCOVA, and *p* was considered significant at < 0.05. Data are available from the Dryad Digital Repository:

## Results

### Animal model

**Fig 2:**
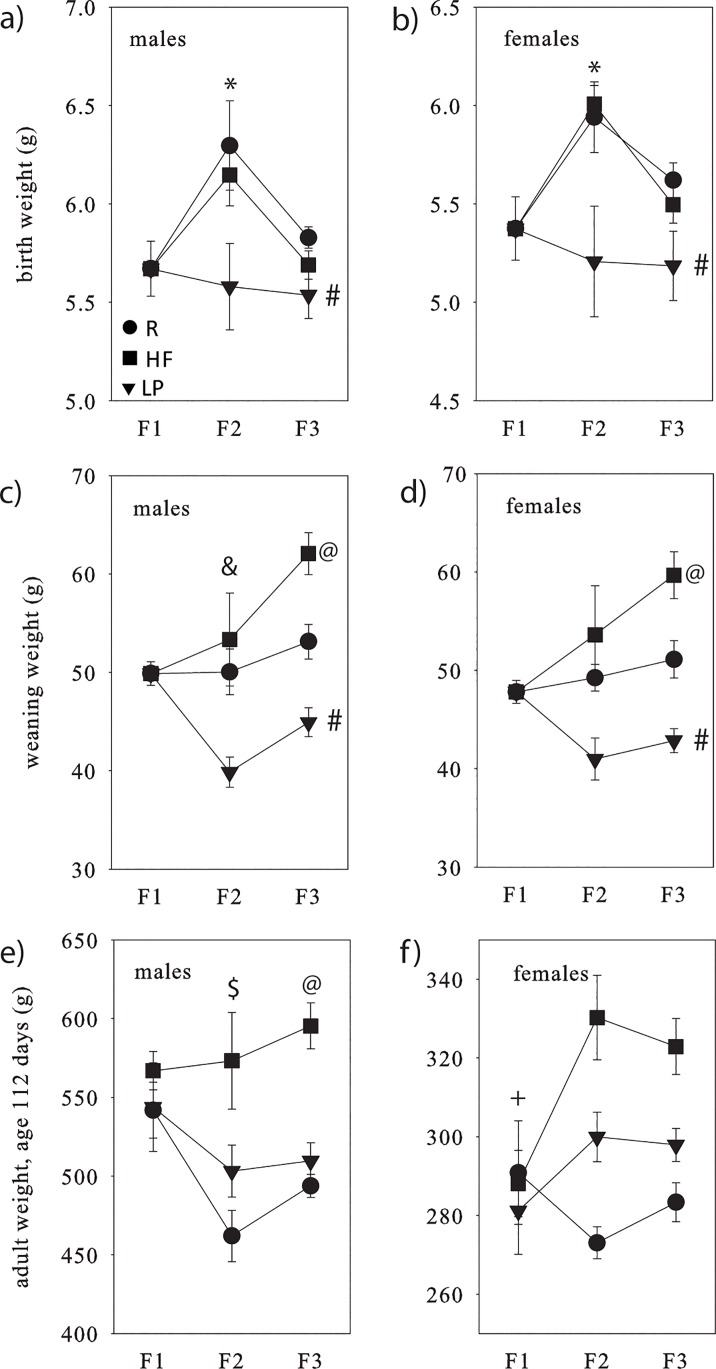
Offspring birth weights (a,b), weaning (c,d) and adult weights (e,f), by sex. Circles represent R, squares represent HF and triangles represent LP. Data are shown as means ± SEM. ^*^F2>F3; ^#^LP<R; ^&^F2<F3; ^@^HF>R and LP; ^$^F1>F2, ^+^ HF:F1<F2. Results of the corresponding statistical analyses are shown in S3 Table.

Details of the animal model including maternal weight gain and food intake during pregnancy, litter size, maternal food intake during lactation are presented in S3 Table and S1 Fig Pup birth weights, weaning and adult weights are presented in Fig 2 and S3 Table. There was an overall higher birthweight in F_2_ compared to F_3_ and birthweights in the LP group were lower than for the R group for both males and females (Fig 2A and 2B). At weaning, body weights at F_2_ were lower in F_2_ males compared to F_3_ but this was not observed in females. For both males and females, weaning weights in the HF group were increased compared to both R and LP groups and the LP group weaning weights significantly lower compared to both R and HF offspring (Fig 2C and 2D). For male adult body weights, F_1_ weights were higher than for F_2_ and HF weights greater than for R (Fig 2E). For adult females, there were no differences across R and LP groups for HF offspring F_1_ weights were reduced overall compared to F_2_ (Fig 2F). As a baseline control, we have also shown in an independent cohort that male and female offspring fed the standard chow control diet do not show any weight changes over successive generations (S2 Fig).

### Body composition

**Fig 3:**
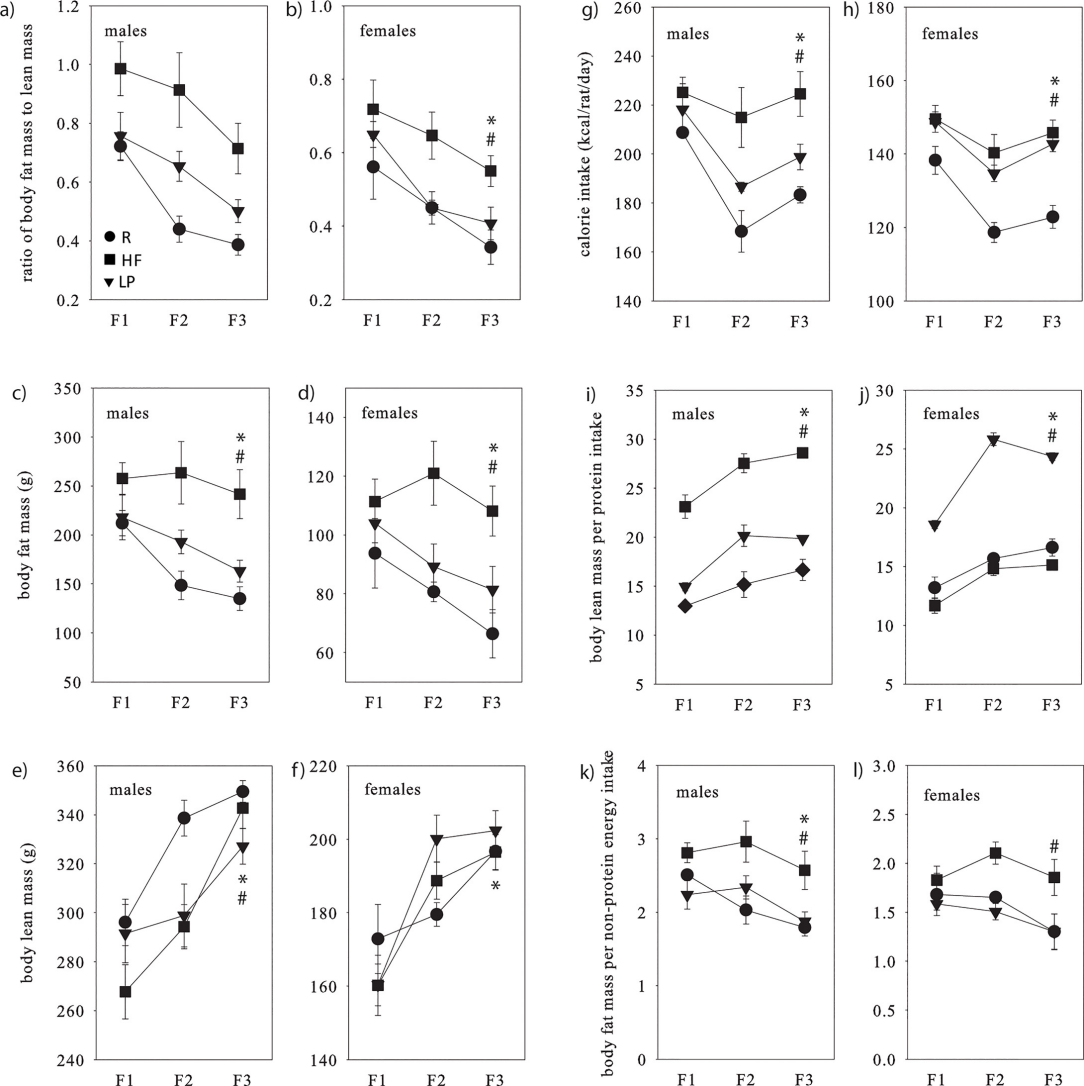
(a-f) Body composition, (g-h) calorie intake and (i-l) nutrient conversion efficiencies. Circles represent R, squares represent HF and triangles represent LP, where body composition and nutrient intake values are means ± SEM, and nutrient conversion efficiency values are estimated marginal means ± SEM. *effect of generation, ^#^effect of dietary group. Results of the corresponding statistical analyses are shown in Table 2.

Fat mass, lean mass and the ratio of fat to lean mass all changed across generations both for males and females (Table 2, Fig 3A–3F). For both sexes, fat mass in F_3_ was reduced relative to F_1_, and lean mass was greater in F_2_ and F_3_ relative to F_1_. In males, lean mass was also greater in F_3_ relative to F_2_. In both sexes, the ratio of fat to lean mass was reduced in F_2_ and F_3_ relative to F_1_. Macronutrient balance did not affect the trajectory of change across generations in any of our three measures of body composition for either sex, but had an effect within generation on fat mass and the ratio of fat to lean mass for both sexes and on lean mass for males. Fat mass and the ratio of fat to lean mass were greater in HF than R for both sexes, and in males only the ratio of fat to lean mass was greater in LP than R and lean mass was reduced in HF and LP compared to R.

**Table 2:** ANOVA results for body composition, calorie intake and food conversion efficiencies.

Sex	Measure	Generationx diet			Generation			Diet		
		*F*	*df*	*p*	*F*	*df*	*p*	*F*	*df*	*p*
**Males**	body fat mass	1.0	4	0.393	5.1	2	0.009	17.6	2	<0.001
	body lean mass	2.0	4	0.990	23.8	2	<0.001	5.8	2	0.005
	body fat: lean	1.0	4	0.423	14.2	2	<0.001	20.6	2	<0.001
	calorie intake	1.8	4	0.140	16.0	2	<0.001	27.4	2	<0.001
	protein conversion eff.	1.6	4	0.208	18.2	2	<0.001	5.1	2	0.012
	non-protein conversion eff.	0.8	4	0.565	7.7	2	0.002	13.7	2	<0.001
**Females**	body fat mass	0.9	4	0.497	3.5	2	0.036	12.3	2	<0.001
	body lean mass	1.9	4	0.127	27.0	2	<0.001	0.6	2	0.555
	body fat: lean	1.3	4	0.283	11.1	2	<0.001	10.0	2	<0.001
	calorie intake	0.7	4	0.593	9.9	2	<0.001	33.7	2	<0.001
	protein conversion eff.	1.1	4	0.366	26.8	2	<0.001	4.6	2	0.019
	non-protein conversion eff.	0.6	4	0.677	2.7	2	0.085	9.6	2	0.001

### Calorie intake

Calorie intake was also non-consistent across generations for both sexes (Table 2, Fig 3G and 3H), with F_2_ and F_3_ consuming fewer calories than F_1_. In females only, F_2_ consumed fewer calories than F_3_. Macronutrient balance did not affect the trajectory of change in food intake across generations for either sex, but did have a within-generation effect for both sexes, where HF and LP consumed more calories than R.

### Food conversion efficiency

The conversion efficiency of ingested protein to body lean mass was non-consistent across generations for both sexes (Table 2, Fig 3I and 3J). In both sexes, this efficiency was greater in F_2_ and F_3_ relative to F_1_, and in males this efficiency was also greater in F_3_ relative to F_2_. Macronutrient balance did not affect the trajectory of change across generations, but did have a sex-dependent effect within generation, where in males HF had reduced efficiency compared to R and in females LP had greater efficiency than R.

The conversion efficiency of ingested carbohydrate and fat to body fat mass was non-consistent across generations for males only, where it was reduced in F_3_ relative to F_2_ (Table 2, Fig 3K and 3L). Macronutrient balance did not affect the trajectory across generations, but had a sex-dependent effect within generation, where in males LP had reduced efficiency compared to R and in females HF had greater efficiency than R.

## Discussion

Based on epidemiological and experimental models of transitory environmental changes, it is probable that perturbing the environment of the parents will increase the risk of chronic disease in first generation offspring by generating an adipogenic phenotype [3]. An important question, however, is whether this phenotype will persist across generations if the environment remains unaltered in the transitioned state? One possibility is that the phenotype might persist across generations if on-going exposure to the transitioned state recreates the same response in each generation. Alternatively, the phenotype may not persist across generations if epigenetic inheritance affects the way successive generations respond to the transitioned environment.

In the present study we demonstrate that following an environmental transition in the parental generation, when successive generations of rat offspring are maintained under constant conditions in the transitioned environment the phenotype is not stable across generations. This instability across generations involves changes in body composition, calorie intake and nutrient conversion efficiencies, all of which are factors in the development of obesity and its co-morbidities. While transgenerational effects on phenotype have previously been reported in offspring following a transitory maternal environmental perturbation [10, 12, 15, 16], this is the first time, to our knowledge, that they have been shown to occur when offspring are maintained in the transitioned environment rather than reverting to pre-transition conditions. This is a critical point of difference for two reasons. First, our design bears more relevance to the human scenario in which populations worldwide are transitioning to and have subsequently continued to experience obesogenic environments. Second, with our design we are able to address the question of whether phenotypes can adapt to being maintained in the transitioned environment [33].

The change in offspring phenotype in this study involved a reduction in adiposity (ratio of fat to lean body tissue) and a reduction in appetite (calorie intake) across successive generations following the parental environmental transition. To interpret this response trajectory, since we were not able to measure the difference in phenotype between the parental generation and their first generation offspring, we refer to the studies of transitory maternal environmental perturbations. These studies, reviewed by [1, 3], highlight an increase in susceptibility to chronic disease in the adult offspring of mothers who experienced an environmental perturbation. Based on this outcome, we can assume that the first generation offspring in the present study had greater susceptibility to nutrition-related, chronic disease relative to animals whose parents did not experience an environmental transition. Therefore, in the present study an increase in adiposity and appetite across successive offspring generations would suggest an exacerbation of this high-risk phenotype, while a reduction in adiposity and appetite across generations would suggest an amelioration of the high-risk phenotype. Our results corresponded with the latter scenario, in which a reduction in adiposity and appetite with time and experience in the transitioned environment represents an amelioration of disease risk.

Interestingly, the trajectories of change in these phenotypes did not differ significantly between the three dietary lineages (reference, high fat and low protein). This suggests that the transgenerational pattern of change was related to the change in environment and/or purified nature of the transitioned diet but independent of the macronutrient balance of the transitioned diet. The respective roles that the change in environment and the change in nutrition played in inducing the initial transgenerational response have yet to be fully elucidated. We can infer that the change in nutrition must have played a role because rats that have relocated from the supplier’s facility to our research facility that are fed a grain-based diet in our facility do not typically become fat and show a consistent growth profile over success generations. It is however possible that nutrition interacted with the relocation-induced stress to exacerbate the impact of the obesogenic diets [34]. Chronic stress is associated with hyperphagia in humans [35, 36], and in migrant populations has been implicated as a factor that interacts with the availability of processed foods to generate obesity [37, 38]. In the rat, it has been shown that mild stress in the presence of food can lead to hyperphagia and obesity [39–41].

The reduction in adiposity across generations shown by rats in this study was the product of both a decline in absolute fat mass and an increase in lean mass. The decline in food intake concomitant with change in body composition accounted for the decline in body fat mass in females, while males also showed a reduction in the conversion efficiency of ingested carbohydrate and fat to body fat mass, possibly achieved by changes in diet-induced thermogenesis [42]. For both sexes, the increase in body lean mass over this same period during which food intake declined suggests a significant increase in the conversion efficiency of ingested protein to body lean mass. Further work is needed to determine how this was achieved and thus whether or not the same is possible in humans. Possible mechanisms that may also apply to humans include changes in the extent and rate of intestinal absorption of dietary protein, or changes in the fraction of the amino acid load that contributes to structural/functional protein needs versus oxidation or gluconeogenesis [43]. Lastly, while the transgenerational pattern of phenotypic change was independent of the macronutrient balance of the transitioned diet, it is worth emphasising that macronutrient balance did elicit within-generation effects. For instance, appetite appeared greater in rats fed the high fat and low protein diets and adiposity was greater in rats fed the high fat diet compared to those fed the reference diet, as may be generally expected [44].

Epigenetic inheritance is the most likely mechanism for the change in phenotype observed across generations in the present study. Due to the timescale of our experiment, the possibility of progressive or reversible changes in DNA methylation or microRNA profiles may be some of the molecular epigenetic factors underlying the trans-generational physiological alterations we observed. An example of this has been reported for fly development whereby a wide range of mechanisms (some epigenetically inherited) were shown to develop to cope with recurrent environmental challenges [45]. A further example was that of recent work by Li *et al*. which showed that a sustained dietary change (excess dietary methyl donors) increased epigenetic variation in isogenic mice [46]. Further, there are also a number of proximate mechanisms that may be involved, such as transmeiotic epigenetic mechanisms, changes to female body condition (e.g. uterus size, hormonal balance etc.) or an aspect of lactation.

A further mechanism that may be involved is changes to the gut microbiota. Gut microbiota has the potential to modulate obesity through rates of fermentation and energy extraction as well as other microbially modulated mechanisms [47]. For example, studies with mice where the gut microbiota of obese mice and lean controls was transplanted into germ-free recipients have shown that mice receiving the gut microbiota from obese donors gained significantly more body fat than the mice receiving the lean microbiota [48–50]. Which proximate mechanisms were involved in the transgenerational response found in this study is an interesting question which may be answered in future independent studies.

If our results do represent a form of adaptation to the transitioned environment, this would align with considerations of developmental plasticity and in particular the concept of predictive adaptive responses (PARs) [51–53]. The PARs hypothesis proposes that the degree of mismatch between the pre- and postnatal environment is a major determinant of subsequent disease. Thus, it is thought that whilst adaptive changes in fetal physiological function may be beneficial for short term survival *in utero*, these changes may be maladaptive in postnatal life and contribute to poor health outcomes particularly when offspring are exposed to catch-up growth, diet-induced obesity and other environmental factors. These so-called PARs therefore are proposed to be not merely side-effects of altered growth *in utero*, but are a strategy designed to optimise the physiology of the offspring for the postnatal environment it is most likely to experience, that is, the ‘predicted’ environment. As an example, suboptimal fetal nutrition may lead to metabolic adaptations which act to maximally utilise limited nutrient availability and therefore increase the chances of survival in continued poor conditions after birth. However these adaptations serve to increase the risk for metabolic disorders when exposed to an enriched postnatal nutrient environment. The risk of chronic disease can therefore be increased in offspring when their anticipated and realised environments are widely discrepant (which may occur if there is a perturbation in the nutritional environment), and their phenotype is inappropriate for the actual environment encountered [51–53]. An extension of this model is that following a permanent change in environmental conditions, experience with the new conditions will accumulate across generations so that the maternal state and thus offspring responses become increasingly appropriate for (and adapted to) the new conditions. The PARS framework also aligns with the work by Vyssotski *et al*. re the notion of epigenetic compensation whereby stresses in one generation can lead to compensatory physiological responses in the next generation [54].

A pressing question is whether and how the work presented here can be translated to the human setting. The nutritional transition applied to the parental generation of rats in our study *i*.*e*. a switch from a grain-based, unrefined diet to a refined-ingredient, high sugar, high fat or low protein diet, is characteristic of the human nutritional transition [55, 56], both in relation to dietary change within societies and the now frequent migration between societies. If successive generations of people were to respond to the transitioned environment in a similar manner to that observed in the present rodent study, this trajectory of change in phenotype would likely represent a shift towards a metabolic phenotype that would present a lower risk of chronic disease in later life.

## Supporting Information

S1 Fig (a) Maternal weight gain and (b) calorie intake in pregnancy, litter size (c) and maternal calorie intake in lactation (d)(not measured for F_1_). Circles represent R, squares represent HF and triangles represent LP, where values are means ± SEM. ^^^F_2_>F_1_; ^%^HF and LP > R. Results of the corresponding statistical analyses are shown in S3 Table.
(EPS)

S2 Fig Body weights in four consecutive generations of male and female rat offspring of normal pregnancies fed a standard chow diet throughout life and housed under standard conditions. n = minimum of 6 litters per group. Data are shown as means ± SEM.
(EPS)

S1 Table Matings, births and neonatal survival. (DOCX)

S2 Table Sample sizes for post-weaning measures. (DOCX)

S3 Table ANOVA results for pregnancy, birth, lactation and offspring weights. (DOCX)
