# Supplementary material for: Successive Generations in a Rat Model Respond Differently to a Constant Obesogenic Environment
Source: PLoS One. 2015 Jul 1;10(7):e0129779. doi: 10.1371/journal.pone.0129779 (PMC4488537; doi:10.1371/journal.pone.0129779)
Supplement: S1 Table — (DOCX) [file pone.0129779.s003.docx]

**S1 Table.** Matings, births and neonatal survival.

| generation mated | diet | no. dams mated | no. dams gave birth | no. litters with 8 or more pups at birth | no. litters with 8 or more pups at day 2 | no. litters with 8 or more pups at weaning |
| --- | --- | --- | --- | --- | --- | --- |
| P | R | 30 | 24 | 24 | 22 | 22 |
| F_1_ | R | 8 | 8 | 6 | 6 | 4 |
|  | HF | 10 | 6 | 5 | 3 | 2 |
|  | LP | 9 | 7 | 7 | 5 | 5 |
| F_2_ | R | 10 | 9 | 8 | 8 | 8 |
|  | HF | 12 | 10 | 9 | 9 | 9 |
|  | LP | 13 | 9 | 8 | 8 | 8 |
