# Supplementary material for: Successive Generations in a Rat Model Respond Differently to a Constant Obesogenic Environment
Source: PLoS One. 2015 Jul 1;10(7):e0129779. doi: 10.1371/journal.pone.0129779 (PMC4488537; doi:10.1371/journal.pone.0129779)
Supplement: S2 Table — (DOCX) [file pone.0129779.s004.docx]

**S2 Table**. Sample sizes for post-weaning measures.

| sex | generation | diet | body weight (no. rats) | body composition (no. rats) | calorie intake (no. cages of 2 rats) |
| --- | --- | --- | --- | --- | --- |
| males | F_1_ | R | 8 | 8 | 4 |
|  |  | HF | 8 | 8 | 4 |
|  |  | LP | 8 | 8 | 4 |
|  | F_2_ | R | 12 | 8 | 6 |
|  |  | HF | 8 | 8 | 4 |
|  |  | LP | 12 | 8 | 6 |
|  | F_3_ | R | 22 | 8 | 11 |
|  |  | HF | 10 | 8 | 5 |
|  |  | LP | 10 | 8 | 5 |
| females | F_1_ | R | 8 | 8 | 4 |
|  |  | HF | 8 | 8 | 4 |
|  |  | LP | 8 | 8 | 4 |
|  | F_2_ | R | 20 | 8 | 10 |
|  |  | HF | 12 | 8 | 6 |
|  |  | LP | 22 | 8 | 11 |
|  | F_3_ | R | 24 | 8 | 12 |
|  |  | HF | 26 | 8 | 13 |
|  |  | LP | 26 | 8 | 13 |
