# Supplementary material for: Successive Generations in a Rat Model Respond Differently to a Constant Obesogenic Environment
Source: PLoS One. 2015 Jul 1;10(7):e0129779. doi: 10.1371/journal.pone.0129779 (PMC4488537; doi:10.1371/journal.pone.0129779)
Supplement: S3 Table — (DOCX) [file pone.0129779.s005.docx]

**S3 Table:** ANOVA results for pregnancy, birth, lactation and offspring weights.

| group | measure | generation*diet | | | generation | | | diet | | | *post-hoc* results |
| --- | --- | --- | --- | --- | --- | --- | --- | --- | --- | --- | --- |
|  |  | *F* | *df* | *p* | *F* | *df* | *p* | *F* | *df* | *p* |  |
| dams | weight gain in pregnancy | 0.2 | 2 | 0.815 | 0.2 | 1 | 0.902 | 2.9 | 2 | 0.069 |  |
|  | pregnancy calorie intake | 1.6 | 2 | 0.237 | 11.0 | 1 | 0.003 | 5.9 | 2 | 0.009 | F_2_ > F_1_; HF & LP > R |
|  | litter size | 1.4 | 2 | 0.218 | 2.6 | 1 | 0.118 | 6.2 | 2 | 0.006 | HF & LP > R |
|  | lactation calorie intake | 0.2 | 2 | 0.808 | 0.4 | 1 | 0.525 | 7.3 | 2 | 0.003 | nil vs. R |
| males | birth weight | 1.2 | 2 | 0.319 | 6.8 | 1 | 0.005 | 5.1 | 2 | 0.013 | F_2_ > F_3_; LP < R |
|  | weaning weight | 0.7 | 2 | 0.486 | 4.7 | 1 | 0.037 | 25.8 | 2 | <0.001 | F_2_ < F_3_; HF > R; LP < R |
|  | adult weight (day 112) | 1.9 | 4 | 0.109 | 19.8 | 2 | 0.019 | 4.4 | 2 | <0.001 | F_1_ > F_2_; HF > R |
| females | birth weight | 1.0 | 2 | 0.382 | 4.3 | 1 | 0.048 | 7.4 | 2 | 0.003 | F_2_ > F_3_; LP < R |
|  | weaning weight | 0.4 | 2 | 0.683 | 2.1 | 1 | 0.156 | 26.4 | 2 | <0.001 | HF > R; LP < R |
|  | adult weight (day 112) | 2.9 | 4 | 0.024 |  |  |  |  |  |  | R & LP: no change; HF: F_1_ < F_2_ |
